# The mediating effect of information sharing on pharmaceutical supply chain integration and operational performance in Ethiopia: an analytical cross-sectional study

**DOI:** 10.1186/s40545-022-00440-0

**Published:** 2022-07-08

**Authors:** Yohannes Birhanu, Tafesse Gizaw, Dawit Teshome, Bekele Boche, Tadesse Gudeta

**Affiliations:** 1 Merawi Hospital, West Gojjam, Amhara Regional State Ethiopia; 2 Last Mile Project, Cordaid Ethiopia, Jimma, Ethiopia; 3 Department of Pharmaceutics and Social Pharmacy, School of Pharmacy, College of Health Sciences, Addis Ababa University, Addis Ababa, Ethiopia; 4 Social and Administrative Pharmacy Unit, School of Pharmacy, Institute of Health, Jimma University, Jimma, Ethiopia

**Keywords:** Information sharing, Supply chain integration, Operational performance, EPSA

## Abstract

**Background:**

Information is crucial in enhancing partnership, reducing uncertainties and inventory costs, improving order fulfillment, and increasing customer satisfaction. However, there is a scantiness of studies on how information sharing affects pharmaceutical supply chain practices and performance. Hence, this study aimed to examine the mediating effect of information sharing between supply chain integration and operational performance.

**Method:**

We conducted an analytical cross-sectional study complemented with a qualitative assessment between May and July 2021. The study populations (*n* = 343) were selected employees working at the Ethiopian pharmaceutical supply agency’s head office and selected hubs. The quantitative data were collected by self-administered five-point Likert-scale questions and analyzed using SPSS^®^-version 23. The mediation effect was determined using sequential linear regression based on the Baron and Kenny stepwise approach. A 95% confidence interval and a p-value less than 5% were used to determine statistical significance. We gathered the qualitative data through in-depth face-to-face interviews with nine key informants and analyzed them using a thematic analysis technique.

**Results:**

Among 320 completed questionnaires returned (with a response rate of 93%), we used 288 in the analysis. Of the respondents, 97 (33.7%) disagreed that information sharing with the agency is simplified. One hundred seventeen (40.6%) disagreed that customers share information via an online system. Most respondents (76.4%) agreed that internal integration in the agency reduced total order time. Information sharing (β = 0.270, *p* < 0.001), customer integration (β = 0.265, *p* < 0.001), and internal integration (β = 0.151, *p* < 0.001) were predictor variables that had a direct positive effect on operational performance. Information sharing posited a partially mediating role between customer integration and operational performance with β = 0.136 at *p* < 0.001. Data quality problems, human-resource-related issues, and natural and human-made calamities were the major challenges affecting information sharing and the overall supply chain practices.

**Conclusion:**

Customer integration, internal integration, and information sharing influenced operational performance positively. Although coordination among the units in the agency is reasonable, there was a lack of communication and quick response from partners, as well as data quality problems and the absence of an automation system in most health facilities. The key informants suggested end-to-end supply systems connections with partners through Enterprise Resource Planning and other means.

**Supplementary Information:**

The online version contains supplementary material available at 10.1186/s40545-022-00440-0.

## Background

A supply chain (SC) is a network of organizations, people, technology, activities, information, and resources that help deliver products from the supplier to the customer [1]. An Integrated Supply Chain (SCI) enhances supplier relationships, core business processes (competencies), and customer expectations, thereby keeping organizations competing more effectively in the market [2]. The SCI is the coordination and alignment of activities within the facilities or among supply chain partners, including suppliers, manufacturers, warehouses, distributors, and retailers, to improve inventory visibility, flexibility, waste reduction, data centralization, and connectivity throughout the entire supply chain [3, 4]. In these processes, information is crucial in achieving a well-integrated supply chain; it improves collaboration with partners, reduces uncertainty, speeds up order fulfillment, lowers inventory costs, and increases customer satisfaction through reliable and timely product delivery [5].

According to the scholars, sharing information benefits SC parties who need to know sales data, forecasts, inventory levels, order status tracking, transportation, customer demand, market status, and a manufacturing production schedule [6–8]. As a result, supply chain integration and information sharing are effective strategies for improving SC performance. The appropriate information must be available at the right time, in the correct form, and by the right person in the right environment to maximize the mutual benefits of the supply chain partners [9–11].

Pharmaceutical supply chain (PSC) activities are integral components of the healthcare system for meeting fundamental customer demands. In today’s highly competitive environment, pharmaceutical organizations must excel at operational performance and identify areas for improvement. Operational performance in the supply chain refers to the activities of the supply chain that meet end-customer requirements for product availability, optimal cost, quality service, delivery reliability, and flexibility in delivering services [12–15]. An organization's overall operational improvements depend on the performance of individual supply chain facilities and their willingness to integrate and collaborate with all supply chain firms [16]. In Nigeria, for example, common challenges within national health care systems that have impacted supply chains include suboptimal estimation, inadequate funding, delays in budgetary allocations, and a lengthy tendering and manufacturing schedule. The National Level Medical Store struggled with distribution planning and vehicle routing due to various factors such as health facility ordering behavior, poor communications and information flow between stakeholders, limited use of technology platforms, poor transportation systems, and vehicle accessibility [17].

Since 2007, Ethiopia has had the Pharmaceuticals Supply Agency (EPSA) mandated to provide a continuous supply of quality-approved, affordable medicines to the public. For efficient operation, the Agency has implemented an Integrated Pharmaceutical Logistics System (IPLS), framework procurement, modernized warehouse management techniques, and optimized delivery routes to health facilities [18]. EPSA has 19 hubs throughout the country, with a head office in Addis Ababa, Ethiopia’s capital, to serve over 4000 health facilities [19]. Despite the efforts and achievements made so far, the supply chain of the country (Ethiopia) faces many obstacles, including long delivery times, frequent stockouts of essential medicines, inadequate skilled human power, intermittent and inaccurate reporting system, lack of top-level management commitment, which in turn lead to inflated product cost, low rate of delivery of goods, and flexibility of the organization [20–26]. Even though such interesting studies addressing various supply chain issues have been conducted from both the EPSA and health facility perspectives, no studies have investigated the role of information sharing in pharmaceutical supply chain integration and organizational performance. Thus, this study aimed to examine the mediating effect of information sharing between supply chain integration and operational performance at an Ethiopian pharmaceutical supply agency.

## Methods

### Study area, design, and period

We conducted an analytical cross-sectional study, accompanied by a qualitative assessment, at the EPSA head office in Addis Ababa and four hubs in different locations. The hubs include Bahir Dar, Gondar, Dessie, and Adama, with the first three being part of the Northwestern cluster of the Amhara regional state. And they are 565, 738, 401, and 96 km, respectively, from the capital. EPSA’s head office had 709 employees, with 168 engaged in pharmaceutical supply chain management. Likewise, the hubs (Bahir Dar, Gondar, Dessie, and Adama) had 608 employees, 175 of whom were involved in pharmaceutical supply chain management. Among the 343 sample frames, 323 were pharmacists, 15 medical laboratory experts, and five biomedical engineers. We collected data between May and July 2021.

### Population and sampling procedure

The source population included all employees at the EPSA head office and selected branches. The study population comprised employees in charge of the pharmaceutical supply chain (PSC) operations at the EPSA head office and selected hubs.

To ensure efficient service provision and coordination, the agency has grouped the 19 hubs into seven clusters based on geographical proximity [19]. In this study, we selected the Northwestern cluster randomly by a lottery method. Among the hubs in this cluster, the Assossa hub was in the area of conflict and instability. As the agency clusters are geographically distant from one another and the investigators' resources were insufficient to perform another random selection, purposive sampling was a viable technique for selecting the replacement cluster. Since the Northwest is a peripheral region, we decided to pick from the mid-cluster. Indeed, we considered the Adama hub along with the head office. We intentionally chose the head office because it connects upstream (manufacturers/suppliers) and downstream supply chain facilities. Three hundred forty-three professionals were involved in PSC operations at the EPSA head office, hubs in the Amhara regional state, and Adama hub. Since this figure was manageable and easy to address, we considered all of them in the study. Concerning the qualitative assessment, we conducted in-depth face-to-face interviews with nine key informants (KIs) chosen based on the position and service experience they had in their agency.

### Data collection procedures

We collected the intended data using structured self-administered questionnaires and interview guides developed through an extensive review of previous literature, including books, journal articles, and reports [7, 27–32].

The questionnaire, which had four parts, was used to collate quantitative data. Part I contained six questions requesting respondents to indicate their socio-demographic information, and part II consisted of four questions about information sharing. Part III was about supply chain integration and measured using 11 questions. Part IV addressed operational performance using ten questions. Parts II–IV used agreement-type questions on a five-point Likert scale marked ‘1’ ‘strongly disagree’ to ‘5’ ‘strongly agree’. We recruited five data collectors (druggists) for the quantitative data collection under the supervision of the investigators. The investigators, on the contrary, collected qualitative data from key informants (KIs) in different departments of the agency. The investigators interviewed the KIs to maintain the consistency of information. The KIs provided written informed consent to audiotape the interview. The interviews were in a local language, Amharic. We asked detailed and probing questions to get broad information on the topic of interest. On average, each interview lasted 30 min.

### Data processing and analysis

The Statistical Package for the Social Sciences, Version 23, was used to enter and analyze quantitative data. The descriptive findings were presented by frequency, mean, and percentages. The inferential statistical analysis was done using sequential linear regression. Before running the final analysis, the assumptions underlying the linear regressions, such as normality, linearity, multicollinearity, and correlations, were checked. The results showed no violation of the underlying assumptions of multivariate statistical analysis. Normality was measured by skewness and kurtosis just after diagnosing and removing outliers by Mahalanobis distance at *p* < 0.001 [33]. For a sample size of 300 or less, the absolute *Z* value of skewness and kurtosis should be less than 3.29 [34]. We inspected the scatter plot of residuals to examine linearity among pairs of variables. Multicollinearity among independent variables was checked by using tolerance and VIF (variance inflation factor) statistics with cut-off points of > 0.2 and < 3, respectively [35].

Before conducting a mediation analysis, it is cardinal to determine whether there is a significant correlation between variables. A Pearson's product–moment correlation was used to measure the magnitude and strength of the linear relationship between variables. The mediation effect of information sharing was determined using the Baron and Kenny stepwise approach with a linear regression [36]. As a result, we iterated regressions and checked the significance of the coefficients at each step. Direct effects were estimated using multivariate linear regression with an independent variable and a mediator predicting the dependent variable while controlling one of the predictors (Additional file 1).

The statistical significance was determined at a 95% confidence interval (CI) and a *p*-value less than 5%. We used the following rules of thumb to determine the degree of mediation. (a) A full mediation occurs when the effect of a direct path is not significant, but becomes significant on an indirect one. (b) There is partial mediation if both the direct and indirect effects are significant; (c) there is no mediation if the direct effect is significant, but the indirect is insignificant [37]. The indirect (mediation) effect level of significance was determined using the web-based Sobel’s test [38].

The qualitative data were analyzed manually using a thematic analysis technique. First, the investigators transcribed and translated the recordings into the English language. A qualitative research expert verified the accuracy of the transcription and translation. Then variables were coded and arranged in a word document in a tabular form, and themes were identified by combining the variables with similar codes. These include data quality issues, human resource and capacity-building, natural and human-made calamities, and PSCI-related issues. Finally, the themes were described in narration form, followed by the quotations of some opinions.

### Data quality assurance

A pre-test was conducted on five percent of Jimma Hub employees to assess the understandability and applicability of the data collection formats. And then, a reliability test was performed, and changes were made to the questions based on the Cronbach’s alpha values, α > 0.7 [31].

The lead investigators trained the data collectors on the objectives and relevance of the study, the protocol, and details concerning participation in the study. Daily, the investigators supervised the data collection process. In the qualitative data, all authors were involved in record transcription, analysis, elucidation, and writing. A senior researcher from Jimma University has checked the transcriptions and translations of the records and interpretations of the findings. Previous experiences and skills aided the investigator in articulating the research questions. The investigators are pharmacists with working experience in health facilities, EPSA, and higher education institutions. Moreover, they have participated in training related to pharmaceutical logistic transactions and IPLS.

## Results

### Socio-demographic characteristics

Three hundred forty-three questionnaires were distributed, with 320 completed questionnaires returned, for a response rate of 93%. After testing the data’s fit to multivariate analysis, 32 outliers were filtered out, leaving 288 for final analysis. The majority of the respondents, 220 (76.4%), were males. Two hundred ten (72.9%) were in the age range of 25–34 years. One hundred seventy-one (59.4%) had first degrees. The majority, 267 (92.7%), were pharmacy professionals. One hundred and sixty-eight (58.3%) had 1–5 years of work experience. Half of the respondents (50%) were from the warehouse and inventory management case team (Table 1).

**Table 1:** Socio-demographic characteristics of respondents (*N* = 288)

Variables		Frequency (%)
Gender	Male	220 (76.4)
	Female	68 (23.6)
	Total	288 (100)
Age	< 25 years	17 (5.9)
	25–34 years	210 (72.9)
	35–44 years	60 (20.8)
	45–54 years	1 (0.3)
	Total	288 (100.0)
Educational background	Diploma	75 (26.0)
	BA/BSC	171(59.4)
	MSc/MA	42 (14.6)
	Total	288 (100.0)
Profession	Pharmacist/druggist	267 (92.7)
	Lab technologist	15 (5.2)
	Biomedical engineer	5 (1.7)
	Others	1 (0.3)
	Total	288 (100.0)
Work experience	1–5 years	168 (58.3)
	6–10 years	111(38.8)
	> 10 years	9 (3.1)
Directorate/case team	Quantification and forecasting	55 (19.1)
	Procurement (tender and contract mgt.)	49 (17.0)
	Warehouse and inventory mgt.*	144 (50.0)
	Distribution and fleet mgt	40 (13.9)
	Total	288 (100.0)

*Management

### Information sharing practices

Among 288 respondents, only 78 (27.1%) agreed that customers’ information sharing with the agency impacts SC responsiveness positively. A substantial proportion of them (33.7%) also disagreed that information sharing with the agency is simplified to improve service quality (Additional file 2).

### Pharmaceuticals supply chain integration

Regarding customer integration, the majority (40.6%) disagreed that customers share information through an online system. Forty-two percent of the participants disagreed that the agency’s distribution occurs at the right time and place. Concerning internal integration, 220 (76.4%) of respondents agreed that internal integration in the agency reduced total order time (Additional file 3).

### Operational performances

The operational performance was measured using cost, quality service, on-time delivery, and flexibility/responsiveness. About 41% of respondents rated a neutral response to the statement that the overall agency’s costs, the logistics, inventory, and operating costs, were kept to a minimum. One hundred fourteen (39.6%) participants agreed that the agency had good customer delivery speed. Only 87 (30.2%) agreed that the agency could quickly adjust and refill unexpected (emergency) needs from customers (Additional file 4).

### Inferential statistical analysis

The following are the results of the basic assumptions of linear regression:

### Normality and linearity test

**Fig. 1:**
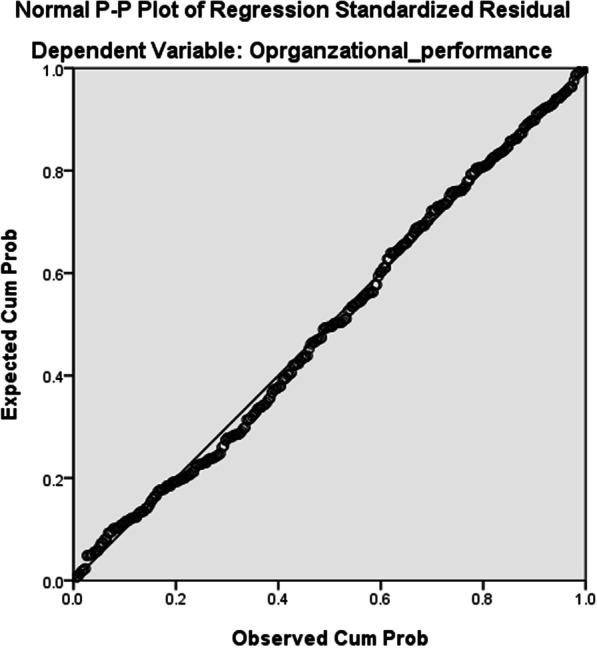
Normal P–P plot standardized residuals (*N* = 288)

A visual examination of the histogram showed a normal distribution of residuals against the predicted dependent variable scores. Additionally, the absolute values of the skewness and kurtosis were between 0.19 and 2.14, and thus, within the accepted threshold value, *Z* < 3.29 (Table 2). Since we removed outliers at the beginning, the scatter plots below showed a linear relationship between the variables (Fig. 1).

**Table 2:** Skewness and Kurtosis value for normality test (*N* = 288)

	Skewness	^a^SE skewness	|Z-skewness |	Kurtosis	SE kurtosis	|Z kurtosis|
Information sharing	0.309	0.144	2.14	− 0.131	0.286	0.46
^b^Cust. integration	0.095	0.144	0.66	− 0.367	0.286	1.28
Internal integration	− 0.194	0.144	1.35	− 0.034	0.286	0.19
^c^Oper. performance	0.242	0.144	1.68	0.104	0.286	0.36

^a^Standard error, ^b^customer integration, ^c^operational performance

### Multicollinearity test

The tolerance (*T*) statistics values for the information sharing, customer integration, and internal integration were 0.728, 0.684, and 0.893, respectively, and were all within the normal range (*T* > 0.2). And the VIF values were calculated to be 1.37, 1.46, and 1.12, respectively, for information sharing, customer integration, and internal integration, and they were all less than three. As a result, there were no issues with multicollinearity.

### Bivariate correlations

The predictors and the mediating variables had a significant positive correlation with the dependent variable (operational performance). Information sharing and customer integration correlated moderately with the dependent variable, with coefficients of 0.440 and 0.454 at *p* < 0.01. The predictors and mediator variables revealed a significant positive correlation with coefficients ranging from 0.216 to 0.519 (Table 3). Thus, conducting a mediation analysis is promising.

**Table 3:** Bivariate correlation among the independent and dependent variables (*N* = 288)

Factors	Information sharing	Customer integration	Internal integration
Information sharing	1		
Customer integration	0.519**	1	
Internal integration	0.216**	0.321**	1
Organizational. performance	0.440**	0.454**	0.295**

**Correlation is significant at the 0.01 level (2-tailed)

### Sequential multiple regression analysis

The model summary of the regression (*R* = 0.533, *p* < 0.001) showed the presence of a significant positive moderate correlation between independent and dependent variables, indicating model fitness (Table 4).

**Table 4:** ^b^Model summary of multiple linear regressions output (*N* = 288)

Model	*R*	*R* square	Adjusted *R* square	Std. error of the estimate	Sig. *F* change
1	0.533^a^	0.284	0.276	0.48246	0.000

^a^Predictors: (Constant), internal integration, information sharing, customer integration

^b^Dependent variable: operational performance

Analysis of variance (ANOVA) also revealed a significant relationship between the dependent and independent variables at *F* (3, 284) = 37.52, *p* < 0.001). Indeed, we rejected the *F*-test for the null hypothesis that none of the predictor variables are related to operational performance (*R* = zero) and concluded that the regression model (with three predictors) significantly predicts the outcome variable (Table 5).

**Table 5:** Significance level for multiple correlation coefficient-ANOVA^a^ (*N* = 288)

Model		Sum of squares	Df	Mean square	*F*	Sig
1	Regression	26.198	3	8.733	37.516	0.000^b^
	Residual	66.107	284	0.233		
	Total	92.305	287			

^a^Dependent variable: operational performance

^b^Predictors: (Constant), internal integration, information sharing, customer integration

### Hypotheses test results

After determining the data’s suitability for multivariate techniques, the hypotheses were tested using simple and multivariate linear regression according to the procedures in the Additional File 1.

*H1: Customer integration positively affects operational performance.* In this study, customer integration had a significant positive direct effect on operational performance with a standardized regression weight (β) = 0.265 at *p* < 0.001. There was also a significant positive total effect on the performance with β = 0.401 at *p* < 0.001. The hypothesis was accepted. On the other hand, operational performance improves with regular review meetings, supportive supervision, and collaborative strategic development with the customer.

*H2: Internal integration positively affects operational performance.* The internal integration had a significant positive direct and total influence on operational performance, with unstandardized regression estimates of 0.151 at *p* = 0.005 and 0.166 at *p* = 0.003, respectively. The hypothesis was accepted. Internal integration substantially improves service quality, order cycle times, customer value, and communication between departments.

*H3: Information sharing positively affects operational performance.* Information sharing influenced operational performance directly with a positive regression value of β = 0.270 at *p* < 0.001. The hypothesis was accepted. The use of the same communication platform, the application of an automated system, and continuous and streamlined information interchange will increase operational performance dramatically.

*H4: Information sharing mediates the relationship between customer integration and operational performance.* The direct and indirect paths were statistically significant, with standardized beta coefficients of 0.265 at *p* < 0.001 and 0.136 at *p* < 0.001, respectively. Thus, information sharing partially mediates customer integration and operational performance.

The hypothesis was accepted.

*H5: Information sharing mediates the relationship between internal integration and operational performance.* Internal integration had a significant positive direct effect at β = 0.151, *p* = 005. Since the indirect path (β = 0.015, *p* = 0.315) was not statistically significant, the hypothesis was not accepted (Table 6).

**Table 6:** Stepwise regression analysis based on the Baron and Kenny approach (*N* = 288)

Steps	Tested path	UStd.c (B)	SE	Std.c (β)	95% CI	Sig
1	Path B: total effect of C int. on OP	0.315	0.043	0.401	[0.230, 0.400]	< 0.001*
	Path D: total effect of II on OP	0.149	0.049	0.166	[0.052, 0.246]	0.003*
2	Path A: effect of C Int. on IS	0.559	0.059	0.502	[0.442, 0.676]	< 0.001*
	Path C: effect of II on IS	0.070	0.068	0.055	[− 0.064, 0.203]	0.303
3	Effect of IS (Path F) on OP	0.191	0.042	0.270	[0.109, 0.273]	< 0.001*
	Effect of IS (Path G) on OP	0.191	0.042	0.270	[0.109, 0.273]	< 0.001*
4	Direct effect of C int. on OP	0.209	0.048	0.265	[0.115, 0.303]	< 0.001*
	Direct effect of II on OP	0.136	0.048	0.151	[0.042, 0.230]	0.005*
5	Indirect effect of C-int. on OP (path A*F	0.107	0.028	0.136	[0.057, 0.167]	< 0.001*
	Indirect effect of II on OP (path C* G)	0.013	0.015	0.015	[-0.014, 0.045]	0.315

*Cint*.-customer integration, *II*-internal integration, *OP*-operational performance, *IS*-information sharing, *SE*-standard error, *UStd.c*- unstandardized coefficients, *Std. c*- standardized coefficients,*significant at *p* < 0.05

### Qualitative findings

We conducted face-to-face interviews with key informants from different agency departments. The participants ranged in age from 31 to 39 years old and had at least 4 years of experience in pharmaceutical logistics and supply chain management. The findings were summarized as follows.

### Data quality issues

Timely information sharing, a complete report, and the availability of an automation system are critical in determining the number of pharmaceuticals forecasted, ordered, safety stock, and meeting the actual demand. Reports such as RRF (Report and Requisition Form), RDF (Revolving Drug Fund), VRF (Vaccine Requisition Form), HCMIS (Health Commodity Management Information System) status, and feedback reports, and other related activities, on the other hand, were exchanged on time. One of the EPSA inventory and warehouse management coordinators highlighted the problem as follows:

*“Responses from different partners on email and telegram communications are not fast enough. The Health Facilities’ (HFs) incomplete reports, inappropriate pharmaceutical quantification, and limited access to automated systems and internet in the down-level facilities result in data quality problems and an information gap between hubs and HFs.” (Team coordinator, eight years of service experience)*

### Human resource and capacity-building-related issues

Building a skilled professional staff is vital to the success of any operation. The lack of qualified professionals is a barrier that results in ineffective operational performance. A well-trained staff is better accustomed to the particulars of their job and will require less or no supervision. Thoughts from this, interviewees mentioned a lack of sufficient training on HCMIS, Dagu (facility edition HCMIS), and other related capacity-building training. As informants revealed, review meetings, workshops, supportive supervision with concerned bodies, and scaling up fill-if initiatives were the best solutions. One of the EPSA forecasting and quantification directorates said the following:

*“There is poor documentation and use of the IPLS format and an interrupted reporting system in healthcare facilities. Due to a lack of training on the Dagu system and other activities, the system has been interrupted in most health facilities. These and other factors, including professional turnover, contribute to the agency’s and health facilities’ service interruptions, difficulty in making decisions, and overstocking/understocking of medicines, resulting in poor operational performance.” (Directorate, 13 years of service experience)*

### Natural and human-made calamities

Uncertainty and emergency conditions like epidemics, pandemics, political instabilities, and social conflicts influence the agency’s daily activities. FMoH and EPSA should collaborate to plan activities before the occurrence and manage such conditions timely. Yet, there were no commonly planned activities before the appearance of such conditions. In the current study, the absence of a standby emergency team affected normal activities and resulted in a slow response to the situations. One of the KIs working under the procurement directorate reported the following:

*“The COVID-19 pandemic and social conflicts in some parts of the country are the main problems contributing to the supply chain disruptions. Some hospitals were designated to treat the army’s wounded, others as centers for COVID-19. Patient visits to healthcare facilities also decreased significantly. Therefore, accessing real-time data during these uncertainties is difficult; Actual use is unknown for a few days leading to reduced consumption and increased wastage (expiry date) of medication in institutions. In addition, there is no organized team for emergency preparedness though reserve pharmaceuticals are available at central EPSA for emergency conditions.” (Directorate, four years of service experience)*

### PSCI-related challenges

Despite agreements between EPSA and customers (both upstream and downstream) to take responsibility and strengthen integration, they do not do so. According to one of the respondents who held the position of deputy manager:

*“There is a transportation problem during pharmaceuticals distribution and supportive supervision due to fuel shortages and long vehicle maintenance times. The partners control the HCMIS administrator password, and the EPSA staff do not have the privilege to maintain it if the system fails. Local and international suppliers/manufacturers do not supply requested pharmaceuticals timely as per the agreement and do not inform us of their supply period and refill quantity, which greatly affects the procurement and distribution of pharmaceuticals to health facilities. Customers at each level are not communicating and exchanging information to increase relations and improve pharmaceutical availability.” (Deputy Manager with nine years of service experience)*

The study also looked at potential solutions for overcoming the overall challenges, intending to improve information sharing, pharmaceutical supply chain integration, and operational performance. Most of the KIs suggested a common communication platform from head office to healthcare facilities (end-to-end communication) and the implementation of the new ERP initiative to increase the visibility of PSC data.

## Discussion

Information sharing and supply chain integration between the buyer and vendor in the supply chain have been considered effective strategies to increase inventory visibility, reduce the bullwhip effect, lead time, and cost, and increase supply chain profit to improve operational performance [10]. Hence, this study aimed to investigate the influence of information sharing between pharmaceuticals' supply chain integration and operational performance.

This study revealed that a significant proportion (33.7%) of the respondents disagreed that information sharing within the agency is simplified to improve service quality. As mentioned in the qualitative assessments, a lack of prompt customer response, an incomplete report, a limited automation system, and internet outages in most health facilities may have influenced information timeliness. The results agree with a study by Tiye and Gudeta [21].

Regarding supply chain integration, the majority (40.6%) disagreed that customers share information via an online system with the agency. The lack of automated and internet access in most service delivery points could be one of the reasons. This result contrasts the previous studies in that an internet-based system improved supplier–customer relationships and strategic alliances [10, 39]. The KIs suggested that system-wide adoption of ERP could improve the customer–supplier relationship. Similarly, 42% of respondents disagreed that the agency’s distribution occurs at the right time and place. However, as the previous study showed, timely and reliable delivery from the upstream partners will improve the delivery performance of the downstream partners [40]. Evidence from key informants revealed that the agency faced a transportation problem, a lack of coordination among customers, and gaps in information exchange to deliver the pharmaceuticals at the right time and in the right place.

Concerning internal integration, 220 (76.4%) of the respondents agreed that the coordination among the units reduced total cycle time. Thus, it is an example of good internal integration and is consistent with the notion of scholars. Internal integration among functional areas reduces the order cycle, improves communication, contributes to innovative project development, and improves customer service [41].

Concerning operational performance, 41% of respondents gave a neutral response to the statement that the logistics, inventory, and operating costs, were kept to a minimum. It might be because the employees were less aware of the situation (the majority having 1–5 years of experience). However, according to the KIs interviews, hard currency inflation, COVID-19 influence, and conflict and instability in various parts of the country have substantially increased the price of pharmaceuticals and overall logistics costs in the last three or four years. Cost directly impacts organizational profitability and performance [30, 42]. On the other hand, only 87 (30.2%) agreed that the Agency quickly adjusts and refills unexpected (emergency) customers' needs. It is a suggestion that the agency needed substantial preparation and flexibility to respond promptly during uncertainty. It is also backed up by the qualitative findings wherein the absence of an emergency team, unplanned conditions, and information gaps became obstacles to the agency. These findings are different from the Sweden and Finnish business reports, where suppliers respond to emergency cases promptly [43, 44]. Supply chains should demonstrate flexibility in the range and volume of products or services they can accommodate to satisfy customers. Improving the flow of information reduces SC uncertainty, speeds up the decision-making process, and enhances collaboration, ultimately leading to better operational performance [45].

From the inferential statistical analysis, both customer and internal integration had a positive significant direct effect on the operational performance with regression weights of β = 0.265 at *p* < 0.001 and β = 0.151 at *p* = 0.005, respectively, supporting H1 and H2. As a result, customer and internal integration improve operational performance through a customer-focused approach and long-term relationships, encouraging inter-organizational integration based on collaborative work between departments and arranging internal and external meetings and supply chain [5]. Flynn et al. and Stank et al. also indicate that supply chain integration influences operational performance [10, 46]. KIs also suggested that a common communication platform, supportive supervision, and training are essential to strengthen supply chain integration and improve operational performances.

The present study revealed that information sharing affected operational performance directly with β = 0.270 at *p* < 0.001 supporting hypothesis 3. Hence, building system-wide communication channels like ERP, real-time response to and from the customer, applying the same communication platforms, and timely feedback system within and out of the agency are recommendations by KIs to improve operational performance. A related study by Liu and Vickery et al. found a positive and direct relationship between information sharing and supply chain performance [47, 48].

Because the direct and indirect pathways were significant with a standardized beta value of 0.265 at *p* < 0.001 and 0.136 at *p* < 0.001, the exchange of information partially mediated customer integration and operational performance, thus confirming hypothesis 4. Accordingly, encouraging accurate demand forecast by the customers, real-time information exchange with customers for determining the effectiveness of stock management and level of uncertainties, and following a collective, integrative, and complementary supply chain structure play pivotal roles in performance improvement. According to Sormaz et al. information sharing is the invaluable focus of strengthening the relationships between customer integration and operation performance [49]. Kokoglu et al. reaffirmed that information exchange is a fundamental supply chain strategy for better logistics and supply chain integration by enhancing cooperation, minimizing uncertainty, increasing material flow, and customer satisfaction through reliable and fast delivery of products [5]. However, as indicated in the in-depth interviews, there were communication gaps across the pharmaceutical supply chain system from the upstream to the downstream facilities. The responses of the various partners by e-mail and telegram are not fast enough. Incomplete reports from healthcare institutions, inadequate quantification of pharmaceuticals, and limited access to automated systems and the internet in lower-level units lead to data quality issues and information gaps between hubs and healthcare facilities. Internal integration, however, had a significant positive direct effect, but the indirect influence was not statistically significant. As a result, we failed to accept the hypothesis. Nonetheless, it does not mean that information sharing is not essential for internal integration. However, it may indicate that information sharing is not a problem within the organization, as evidenced by the descriptive findings, in which 220 (76.4%) of respondents agreed that coordination among the units is reasonable.

### Limitations of the study

No similar primary research was available to validate the proposed framework, and we used a few non-health articles to develop the conceptual framework. Most EPSA hubs were not part of the study due to time, budget, and human resource constraints. The study participants were the staff of EPSA alone, and future researchers can study from the perspective of customers and suppliers. To replace the excluded hub in the risk area, the researchers purposely chose the middle cluster; this can limit the generalizability of the results.

## Conclusion

The results showed that customer integration, internal integration, and information sharing influenced operational performance significantly and positively. According to the hypotheses test results, information sharing partially mediates the relationship between customer integration and operational performance with a significant positive standard beta coefficient. Although the agency’s internal integration was reasonably good, there was a lack of communication and quick response from partners, a lack of common communication platforms, the absence of an automation system in most health facilities, and skill gaps. The key informants also recommended implementing an end-to-end connection with partners through ERP and other means.

## Supplementary Information

**Additional file 1:** The Baron and Kenny stepwise approach.

**Additional file 2:** Information sharing practice at EPSA (*N* = 288).

**Additional file 3:** Pharmaceutical supply chain integration practice of EPSA (*N* = 288).

**Additional file 4:** Operational performance of EPSA (*N* = 288).

## Data Availability

The data sets generated during and/or analyzed during the current study are available from the corresponding author on reasonable request.

## Abbreviations

EPSA: Ethiopian Pharmaceutical Supply Agency
ERP: Enterprise Resource Planning
FMoH: Federal Ministry of Health
HCMIS: Health Commodity Management Information System
IPLS: Integrated Pharmaceutical Logistics System
KIs: Key informants
NGOs: Non-governmental organizations
PSC: Pharmaceutical supply chain
PSCI: Pharmaceutical supply chain integration
SC: Supply chain
SCI: Supply chain integration
