# Supplementary material for: The mediating effect of information sharing on pharmaceutical supply chain integration and operational performance in Ethiopia: an analytical cross-sectional study
Source: J Pharm Policy Pract. 2022 Jul 8;15:44. doi: 10.1186/s40545-022-00440-0 (PMC9264740; doi:10.1186/s40545-022-00440-0)
Supplement: Supplementary file 1 — Additional file 1: The Baron and Kenny stepwise approach. [file 40545_2022_440_MOESM1_ESM.docx]

**The Baron and Kenny stepwise approach**

(a) Total effects path B and D (bivariate linear regression) (Fig 1)


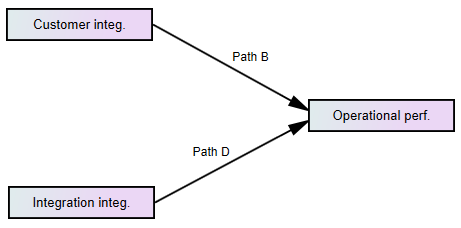


Figure 1. Total effects of customer integration and internal integration

(b) Direct (multivariate linear regression) and indirect (Path A x Path F and Path C x Path G) (Fig 2 and 3)

| 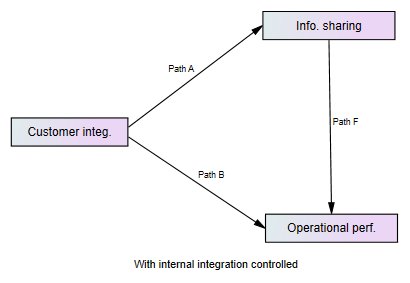  Figure 2. Mediation effects of information sharing between customer integration and operational performance | 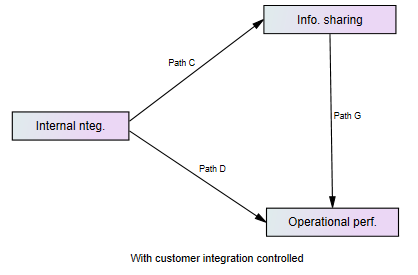  Figure 3. Mediation effects of information sharing between internal integration and operational performance |
| --- | --- |

**Steps to evaluate mediation effects**

Step 1: Conduct a bivariate leaner regression analysis with each predictor variables (X) predicting operational performance to determine total effects of each relation

- Y=β_o_+β_1_X+e…………………. where “X” represents customer integration or internal integration

Step 2: Run bivariate linear regression with X predicting M to determine path A and C while controlling one of the predictors in both Fig 2 and Fig 3.

- M= β_o_+β_1_X+e……………...where M is information sharing

Step 3: Conduct a multivariate regression analysis with “X” and “M” predicting operational performance to calculate path F and G with each predictor

- Y=β_o_+ β_1_X+ β_2_M+e

Step 4: Conduct a multiple linear regression analysis with “X” and “M” predicting operational performance to determine the direct effects of the predictors.

- Y=β_o_+ β_1_X+ β_2_M+e
