# Supplementary material for: The mediating effect of information sharing on pharmaceutical supply chain integration and operational performance in Ethiopia: an analytical cross-sectional study
Source: J Pharm Policy Pract. 2022 Jul 8;15:44. doi: 10.1186/s40545-022-00440-0 (PMC9264740; doi:10.1186/s40545-022-00440-0)
Supplement: Supplementary file 2 — Additional file 2: Information sharing practice at EPSA (N = 288). [file 40545_2022_440_MOESM2_ESM.docx]

Additional file 2. Information sharing practice at EPSA (N=288)

| Information sharing practice | Level of agreement or disagreement | | | | | |
| --- | --- | --- | --- | --- | --- | --- |
| Questions/statements  Customer’s information sharing with the agency positively impacts SC responsiveness | SD (%) | D (%) | N (%) | A (%) | SA (%) | x̄ |
|  | 10  (3.5) | 64 (22.2) | 119 (41.3) | 78 (27.1) | 17 (5.9) | 3.10 |
| Customer’s information sharing with the agency affects delivery speed | 20 (6.9) | 71 (24.7) | 105 (36.5) | 74 (25.7) | 18 (6.3) | 3.00 |
| Information sharing within the agency is a streamlined to improve service quality | 23 (8.0) | 97 (33.7) | 103 (35.8) | 52 (18.1) | 13 (4.5) | 2.77 |
| The quality of information shared with the customers affected the logistics costs | 23 (8.0) | 96 (33.3) | 111 (38.5) | 43 (14.9) | 15 (5.2) | 2.76 |
| Overall mean | | | | | | 2.91 |
| Note: SD-strongly disagree, D-disagree, N-neutral, A-agree, SA-strongly agree | | | | | |  |
