# Supplementary material for: The mediating effect of information sharing on pharmaceutical supply chain integration and operational performance in Ethiopia: an analytical cross-sectional study
Source: J Pharm Policy Pract. 2022 Jul 8;15:44. doi: 10.1186/s40545-022-00440-0 (PMC9264740; doi:10.1186/s40545-022-00440-0)
Supplement: Supplementary file 3 — Additional file 3: Pharmaceutical supply chain integration practice of EPSA (N = 288). [file 40545_2022_440_MOESM3_ESM.docx]

Additional file 3. Pharmaceutical supply chain integration practice of EPSA, (N=288)

| Customer integration | Level of agreement or disagreement | | | | | | |  |
| --- | --- | --- | --- | --- | --- | --- | --- | --- |
| Questions/statements | SD (%) | D (%) | N (%) | | A (%) | SA (%) | | x̄ |
| Customers with agency integration make decisions that are mutually beneficial. | 11 (3.8) | 75 (26.0) | 109 (37.8) | | 83 (28.8) | 10  (3.5) | | 3.02 |
| Agency invest information sharing with customers if the outcome is immediate | 9 (3.1) | 82 (28.5) | 111 (38.5) | | 80 (27.8) | 6 (2.1) | | 2.97 |
| Customers share information with agency via online system. | 21 (7.3) | 96 (33.3) | 97 (33.7) | | 68 (23.6) | 6 (2.1) | | 2.80 |
| The Agency distribution is made at the right time and place due to integration. | 38 (13.2) | 83 (28.8) | 98 (34.0) | | 62 (21.5) | 7 (2.4) | | 2.71 |
| With an integrated customers supply chain; logistics services are improved. | 33  (11.5) | 84 (29.2) | 83 (28.8) | | 72 (25.0) | 16 (5.6) | | 2.84 |
| Orders are easily processed as a result of customer integration. | 18  (6.3) | 93 (32.3) | 93 (32.3) | | 77 (26.7) | 7 (2.4) | | 2.87 |
| Overall mean | | | | | | | | 2.87 |
| Internal integration |  | | | | | | |  |
| Questions/statements | SD (%) | D (%) | | N (%) | A (%) | | SA (%) | x̄ |
| Supply chain integration has assisted in improving the quality of goods, works and services offered to the beneficiary. | - | 39 (13.5) | | 76 (26.4) | 129 (44.8) | | 44 (15.3) | 3.62 |
| Customer integration has enabled to deliver services easily and quickly. | 2 (0.7) | 53 (18.4) | | 113 (39.2) | 106 (36.8) | | 14 (4.9) | 3.27 |
| Internal integration among the unit reduces total cycle time | - | 16 (5.6) | | 52 (18.1) | 164 (56.9) | | 56 (19.4) | 3.90 |
| Internal integration reduces total logistics costs | 2 (0.7) | 29 (10.1) | | 116 (40.3) | 125 (43.4) | | 16 (5.6) | 3.43 |
| Internal integration enhance value to the customer | 4 (1.4) | 31 (10.8) | | 111 (38.5) | 131 (45.5) | | 11 (3.8) | 3.40 |
| Overall mean | | | | | | | | 3.52 |
| Note: SD-strongly disagree, D-disagree, N-neutral, A-agree, SA-strongly agree | | | | | | | | |
