# Supplementary material for: The mediating effect of information sharing on pharmaceutical supply chain integration and operational performance in Ethiopia: an analytical cross-sectional study
Source: J Pharm Policy Pract. 2022 Jul 8;15:44. doi: 10.1186/s40545-022-00440-0 (PMC9264740; doi:10.1186/s40545-022-00440-0)
Supplement: Supplementary file 4 — Additional file 4: Operational performance of EPSA (N = 288). [file 40545_2022_440_MOESM4_ESM.docx]

Additional file 4. Operational performance of EPSA (N=288)

| Cost | Level of agreement or disagreement | | | | | | | x̄ |
| --- | --- | --- | --- | --- | --- | --- | --- | --- |
| Questions/statements  The Agency logistics costs are kept at a minimum level | SD (%) | D (%) | N (%) | | A (%) | | SA (%) |  |
|  | 5  (1.7) | 74 (25.7) | 121 (42.0) | | 76 (26.4) | | 12 (4.2) | 3.06 |
| The Agency inventory costs are kept at a minimum level | 6 (2.1) | 82 (28.5) | 127 (44.1) | | 64 (22.2) | | 9 (3.1) | 2.96 |
| The Agency operation costs are kept at a minimum level | 11 (3.8) | 83 (28.8) | 118 (41) | | 71 (24.7) | | 5 (1.7) | 2.92 |
| Overall mean | | | | | | | | 2.98 |
| Quality |  | | | | | | |  |
| Questions/statements | SD (%) | D (%) | N (%) | A (%) | | SA (%) | | x̄ |
| The Agency provide better quality service | 3 (1.0) | 32 (11.1) | 109 (37.8) | 120 (41.7) | | 24 (8.3) | | 3.45 |
| Our suppliers’ pharmaceuticals have good quality | 2 (0.7) | 41 (14.2) | 117 (40.6) | 110 (38.2) | | 18 (6.3) | | 3.35 |
| Product damages/loss on arrival is very low | 2 (0.7) | 48 (16.7) | 110 (38.2) | 106 (36.8) | | 22 (7.6) | | 3.34 |
| Overall mean | | | | | | | | 3.38 |
| Delivery |  | | | | | | |  |
| Questions/statements | SD (%) | D (%) | N (%) | A (%) | | SA (%) | | x̄ |
| The Agency has an excellent on-time delivery record to customers. | 5 (1.7) | 41 (14.2) | 114 (39.6) | 114 (39.6) | | 14 (4.9) | | 3.32 |
| Our partners’ deliveries are reliable and accurate | 5 (1.7) | 73 (25.3) | 117 (40.6) | 83 (28.8) | | 10 (3.5) | | 3.07 |
| Overall mean | | | | | | | | 3.20 |
| Flexibility |  | | | | | | |  |
| Questions/statements | SD (%) | D (%) | N (%) | A (%) | | SA (%) | | x̄ |
| The Agency can quickly introduce new products into the market with the existing system | 1  (0.7) | 66 (22.9) | 128 (44.4) | 75 (26.0) | | 18 (6.3) | | 3.15 |
| The Agency can quickly adjust and refill unexpected (emergency) need from customers | 5 (1.7) | 71 (24.7) | 106 (36.8) | 87 (30.2) | | 19 (6.6) | | 3.15 |
| Overall mean | | | | | | | | 3.15 |
| Note: SD-strongly disagree, D-disagree, N-neutral, A-agree, SA-strongly agree | | | | | | | |  |
